# Unusual Late Presentation of Capsular Bag Distension Syndrome Associated With Propionibacterium acnes Endophthalmitis

**DOI:** 10.7759/cureus.19684

**Published:** 2021-11-17

**Authors:** Abdullah H Al-Mulla, Muath W Al-Rushoud

**Affiliations:** 1 Department of Ophthalmology, Dhahran Eye Specialist Hospital, Dhahran, SAU

**Keywords:** pseudophakia, endophthalmitis, propionibacterium acnes, capsular block, capsular bag distension

## Abstract

This report describes an unusually delayed presentation of capsular bag distension syndrome (CBDS), which was found to be associated with Propionibacterium acnes (P. acnes) endophthalmitis. Our patient presented with a gradual decrease in vision after uneventful cataract surgery done 13 years back. On examination, there was a thick turbid fluid entrapped behind the intraocular lens (IOL). Ultrasound biomicroscopy (UBM) confirmed the presumed diagnosis. The case was managed by pars plana vitrectomy (PPV) with posterior capsulotomy, and the entrapped turbid fluid was aspirated and sent for histopathology, which revealed a positive growth of P. acnes. The patient had excellent outcomes with complete resolution post-operatively.

## Introduction

Capsular bag distension syndrome (CBDS), or capsular block syndrome (CBS), is one of the rare complications of cataract extraction by phacoemulsification technique [1]. It occurs when there are adhesions between the edge of the lens capsule and the optic of the intraocular lens (IOL), which leads to fluid entrapment behind the IOL [2]. Making a small continuous curvilinear capsulorhexis (CCC) of less than 5.5 mm, or leaving a residual cortical lens material has been shown to increase the risk of this fluid entrapment [3]. Clinically, the fluid may appear clear, creamy white, or turbid, depending on the time onset of CBDS, which has been described as late as 14 years following cataract surgery [4]. Late-onset CBDS is usually associated with the turbid type, which can be contagious with an anaerobic gram-positive bacillus, Propionibacterium acnes (P. acnes) [5]. Spreading of this organism can occur after Neodymium-doped yttrium garnet (Nd:YAG) laser posterior capsulotomy [6]. Therefore, it is sometimes better to be avoided and go for a surgical approach. In this report, we will describe the unusual late presentation of CBDS presented 13 years post-cataract surgery which was associated with P. acnes endophthalmitis, which had been managed surgically.

## Case presentation

**Figure 1:**
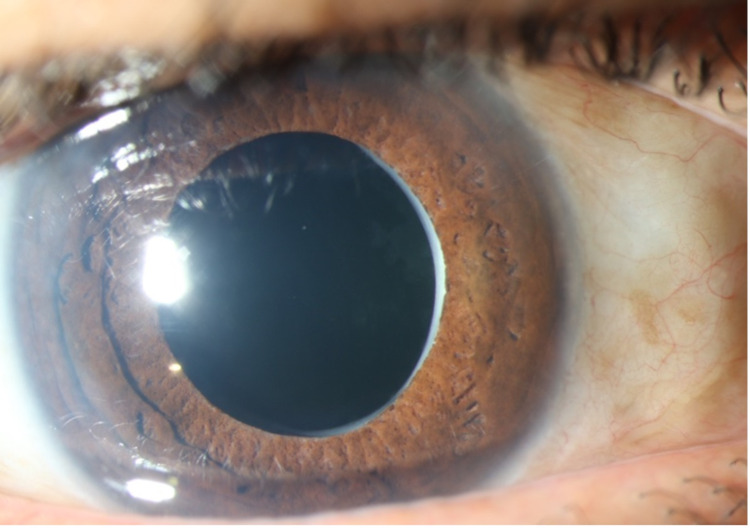
Slit-lamp photograph of the right eye showing a clear intraocular lens (IOL) without any fluid entrapment.

**Figure 2:**
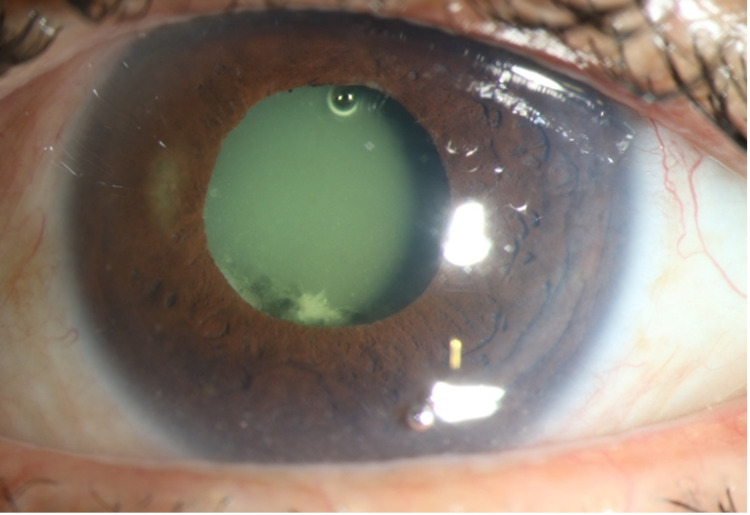
Slit-lamp photograph of the left eye showing a densely turbid fluid entrapped behind the intraocular lens (IOL).

**Figure 3:**
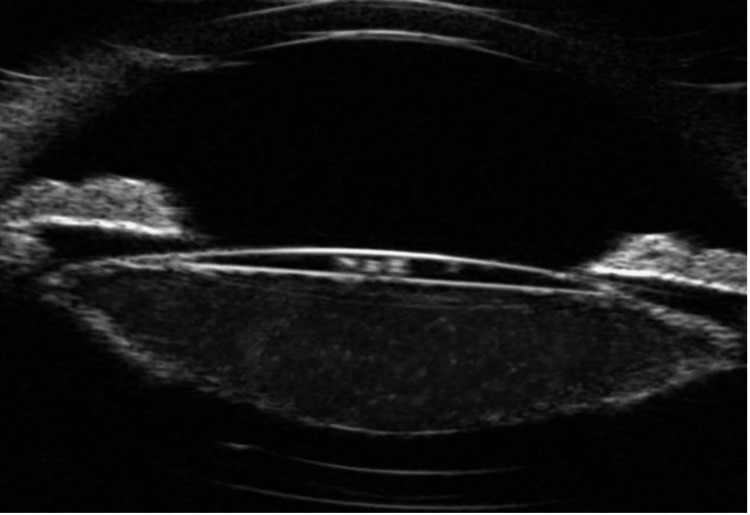
Ultrasound biomicroscopy (UBM) of the left eye showing a distended capsular bag with a hyperechoic collection of turbid fluid pushing the intraocular lens (IOL) forward.

**Figure 4:**
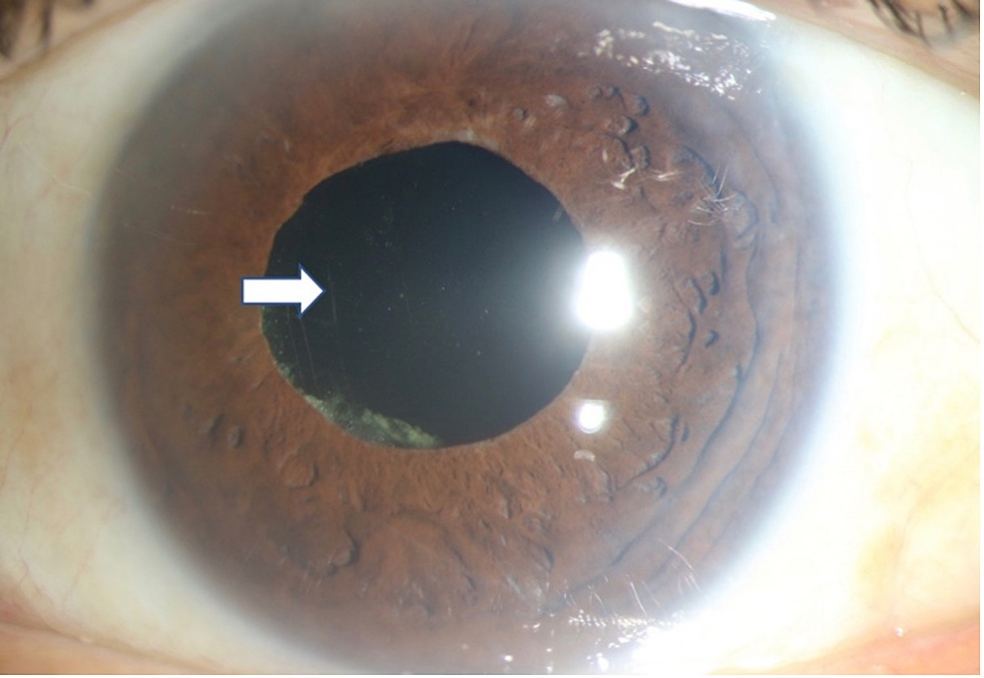
Postoperative photograph of the left eye showing complete resolution of the entrapped fluid with opened posterior capsule. The edge of the posterior capsulotomy can be seen (white arrow).

A 54-year-old medically free Saudi gentleman presented to our clinic complaining of a gradual painless decrease in vision of his left eye over one year. He also reported mild pain, photophobia, and redness from time to time. The patient underwent uneventful cataract surgery of both eyes 13 years prior to his presentation. The best-corrected visual acuity (BCVA) using a Snellen chart was 20/28 in the right eye (OD) and 20/400 in the left eye (OS). Slit-lamp biomicroscopic examination of the OD was unremarkable (Figure 1). Upon examining the OS, there were 1+ (i.e., 6-15 cells per field) mixed anterior chamber cells. CCC was relatively small (measured 4.7 mm). In addition, a thick turbid greenish fluid was noted behind the IOL, limiting visualization of the posterior capsule (Figure 2). B scan was performed due to poor visualization of the fundus, which revealed moderately dense vitreous opacities suggestive of vitritis. There was no associated choroidal thickening. Ultrasound biomicroscopy (UBM) showed a hyperechoic collection of turbid fluid behind the IOL with a distended capsular bag, confirming the presumed diagnosis of CBDS (Figure 3). Late-onset CBDS with a high possibility of associated P. acnes was assumed, and surgical intervention was considered. After explaining the surgical procedure and obtaining written informed consent from the patient, a pars plana vitrectomy (PPV) with a posterior capsulotomy was performed. Initially, 23 gauge three PPV trocars were placed 3.5 mm away from the limbus. Then, through the superonasal sclerotomy site, a 27-gauge needle was inserted to pierce the posterior capsule and gently aspirate the liquified material. After aspirating and clearing the visual axis, a vitreous cutter was introduced through the same superonasal port to create posterior capsulotomy followed by core vitrectomy. Intravitreal vancomycin (1 mg/0.1 mL) was injected at the end of the procedure due to the high possibility of associated P. acnes endophthalmitis. After the procedure, the aspirated material was sent to histopathology for gram stains and cultures. After 72 hours, Thioglycolate broth was positive for the growth of P. acnes confirming the presumed diagnosis. The post-operative period was uneventful, and no signs of intraocular inflammation were detected. At six months, the patient had 20/25 BCVA and did fine without any complaints (Figure 4). 

## Discussion

Capsular bag distension syndrome (CBDS) has been classified based on the time of onset by Miyake et al. as intraoperative, early postoperative, and late postoperative CBDS. The intraoperative type occurs when the balance saline solution (BSS) entraps between the nucleus and the posterior capsular bag during hydrodissection maneuver, which might lead to posterior capsular rupture (PCR) and drop of the nucleus into the vitreous cavity. The early post-operative type (original CBDS) occurs within the first 15 days after the surgery, and it is mainly due to the accumulation of Ophthalmic Viscoelastic Device (OVD) between the IOL and the posterior capsular bag. The third CBDS type, late-onset post-operative, usually occurs months or years after the cataract surgery, with a mean incidence of 3.8 years, and it is mainly because of proliferative and metaplastic changes of residual epithelial and cortical cells which explain the appearance of the thick milky turbid fluid (lacteocrumenasia) in this type [7].

According to this classification, our presenting case is considered a type 3 CBDS and it has three unique features. First, having the late presentation 13 years after uneventful cataract surgery. Second, surprising BCVA (20/400) compared to the patient's clinical picture of having dense turbid fluid with an absent red reflex. And third, having the associated symptoms of recurrent mild pain, photophobia, and redness with the dramatic resolution of these symptoms after the surgical management. To the best of the author's knowledge, up to date, there is only one reported case that described CBDS development after 14 years [4], and only two described it after 13 years (as in our patient) [8,9]. Upon careful examination of the anterior segment with maximal dilation, it was noted that the capsulorhexis of the OS was small (4.7 mm) compared to the OD (5.5 mm), which explains the unilateral involvement in our case. Therefore, it is important to make enough sized CCC during cataract surgery to lower the risk of developing this complication.

The surgical approach by PPV with posterior capsulotomy was the preferred choice for our patient because of two reasons. First, we were unable to visualize the posterior capsule, making it difficult to perform the Nd:YAG laser posterior capsulotomy. And second, there was a high suspicion of associated P. acnes; therefore, violation of the posterior capsule by Nd:YAG laser might cause further dissemination of the infection into the vitreous cavity [5]. Raina et al. and Galvin et al. managed their late-onset CBDS cases by PPV with posterior capsulotomy, similar to our surgical approach, and all cases showed excellent outcomes with complete resolution [9,10]. This emphasizes the good prognosis of this condition with the surgical management by PPV with posterior capsulotomy. However, apart from the well-known complications of this surgical approach, it is considered a more invasive and costly procedure [9].

## Conclusions

To conclude, we presented an unusual late presentation of CBDS associated with P. acnes endophthalmitis. Our case highlights the importance of considering this condition even years after uneventful cataract surgery. In most of the CBDS cases, Nd:YAG laser posterior capsulotomy is a safe technique to relieve this rare condition. However, the concomitant presence of P. acnes should be kept in mind. So, if there is a high suspicion of this infective organism, the surgical approach should be the choice, and Nd:YAG laser posterior capsulotomy is better to be avoided in such cases.
